# Association of Cadherin-Related Family Member 1 with Traumatic Brain Injury

**DOI:** 10.1007/s10571-024-01476-3

**Published:** 2024-04-24

**Authors:** Yong’An Jiang, Peng Chen, YangYang Zhao, Yan Zhang

**Affiliations:** 1 Department of Neurosurgery, The Second Affiliated Hospital, Jiangxi Medical College, Nanchang University, Nanchang, 330006 Jiangxi People’s Republic of China; 2 Nanchang University, Nanchang, 330006 Jiangxi People’s Republic of China

**Keywords:** Cadherin-related family member 1 (CDHR1), Genome-wide association study (GWAS), Quantitative trait loci (eQTL), Traumatic brain injury (TBI)

## Abstract

The cadherin family plays a pivotal role in orchestrating synapse formation in the central nervous system. Cadherin-related family member 1 (CDHR1) is a photoreceptor-specific calmodulin belonging to the expansive cadherin superfamily. However, its role in traumatic brain injury (TBI) remains largely unknown. CDHR1 expression across various brain tissue sites was analyzed using the GSE104687 dataset. Employing a summary-data-based Mendelian Randomization (SMR) approach, integrated analyses were performed by amalgamating genome-wide association study abstracts from TBI with public data on expressed quantitative trait loci and DNA methylation QTL from both blood and diverse brain tissues. CDHR1 expression and localization in different brain tissues were meticulously delineated using western blotting, immunohistochemistry, and enzyme-linked immunosorbent assay. CDHR1 expression was consistently elevated in the TBI group compared to that in the sham group across multiple tissues. The inflammatory response emerged as a crucial biological mechanism, and pro-inflammatory and anti-inflammatory factors were not expressed in either group. Integrated SMR analyses encompassing both blood and brain tissues substantiated the heightened CDHR1 expression profiles, with methylation modifications emerging as potential contributing factors for increased TBI risk. This was corroborated by western blotting and immunohistochemistry, confirming augmented CDHR1 expression following TBI. This multi-omics-based genetic association study highlights the elevated TBI risk associated with CDHR1 expression coupled with putative methylation modifications. These findings provide compelling evidence for future targeted investigations and offer promising avenues for developing interventional therapies for TBI.

**Graphical Abstract:**

Cadherin-related family member 1 as a potential risk factor for traumatic brain injury.

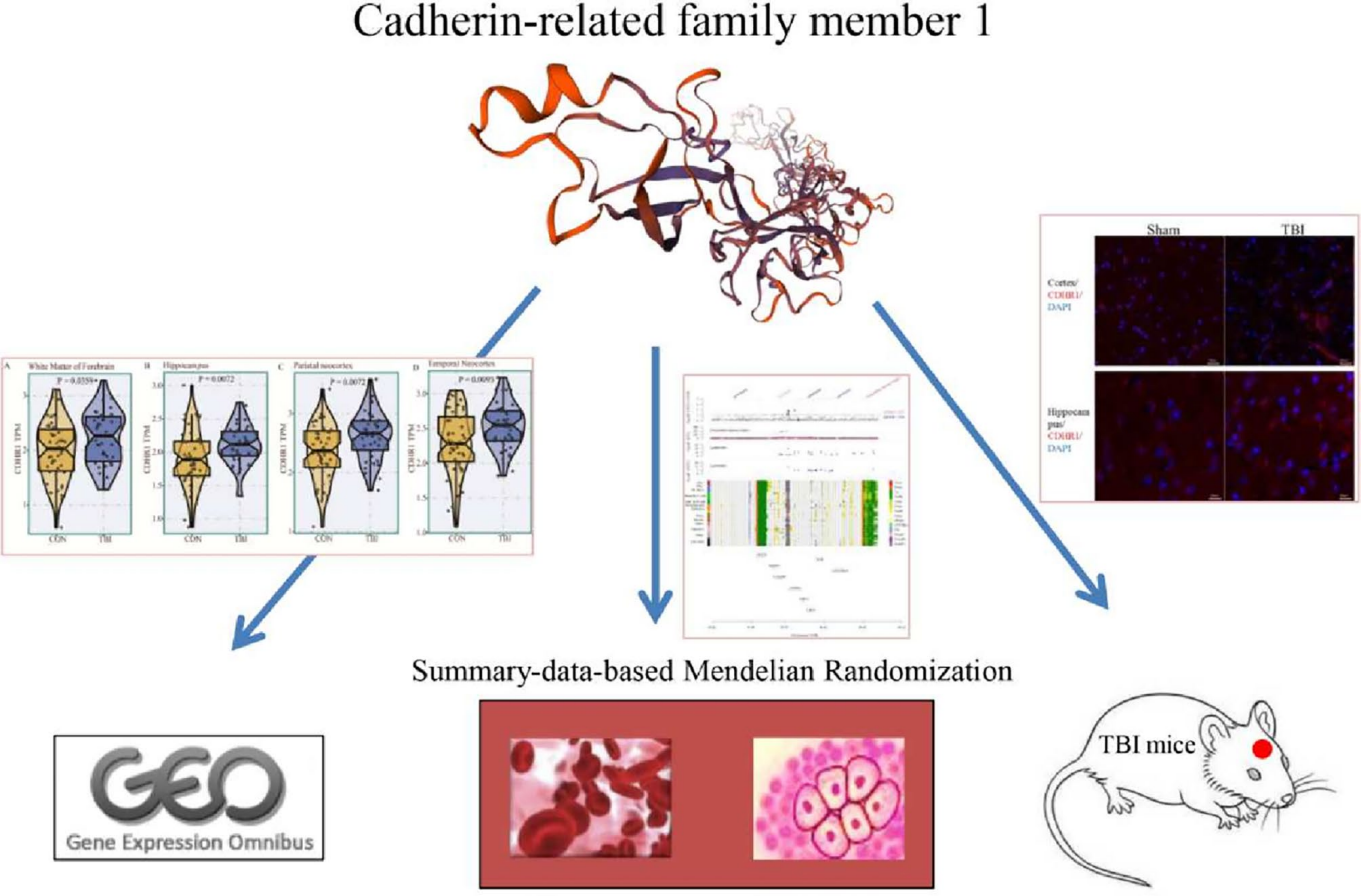

**Supplementary Information:**

The online version contains supplementary material available at 10.1007/s10571-024-01476-3.

## Introduction

Traumatic brain injury (TBI) contributes significantly to patient mortality and disability, affecting a multitude of individuals across the United States and accounting for 7% of all deaths (Liu et al. 2022). Current therapies employed in the treatment and clinical management of patients with TBI often involve invasive procedures such as high-depth therapy, cerebrospinal fluid drainage, and cranial decompression, frequently resulting in severe complications and neurological deficits (Capizzi et al. 2020; Vella et al. 2017; Stocchetti et al. 2017). The heterogeneity observed in individual patients with TBI can be attributed to factors such as age, susceptibility, caregiving approach, and injury severity (Jha et al. 2021; Kals et al. 2022). In addition, molecular regulatory pathways play a pivotal role in augmenting or diminishing the risk of TBI.

Within the realm of molecular investigations, the cadherin superfamily, which comprises transmembrane proteins with repetitive extracellular structural domains known as cadherins, is important. These proteins are integral to the developmental processes of the nervous system, including neurulation, neuronal differentiation and migration, axon growth, pathfinding, target recognition, and synapse formation (Hirano and Takeichi 2012; Yusuf et al. 2022; Blecharz et al. 2008). Of particular interest is cadherin-related family member 1 (CDHR1), a photoreceptor-specific cadherin belonging to the calcineurin superfamily that is characterized by six extracellular cadherin (EC) repeat sequences (Bolz et al. 2005). CDHR1 initially drew attention due to its association with retinal dysplasia (Malechka et al. 2022). CDHR1 has been primarily studied in vision science because of its crucial role in the junctions between the inner and outer segments (Burgoyne et al. 2015; Farag et al. 2023). However, there are limited data describing the expression, distribution, and function of CDHR1 in the brain. Moreover, most studies of CDHR1 in the brain have been related to gliomas, where downregulation of the protein is an unfavorable prognostic factor, and its overexpression prevents glioma growth and invasion (Wang et al. 2021). However, its involvement in neurological disorders, particularly traumatic brain injury, remains unexplored.

To our knowledge, this is the first study to reveal the expression and distribution patterns of CDHR1 in various brain tissues, including the cortex, hippocampus, and cerebellum, of TBI-afflicted mice. Using the Summary-data-based Mendelian Randomization (SMR) method, we postulated a causal link between CDHR1 and TBI from a multiomics perspective. These in vivo experiments substantiate our findings. Notably, our study sheds light on the potential influence of genetic variations within CDHR1, thereby elucidating the mechanisms underlying the effects of TBI.

## Materials and Methods

### Exploration of the GEO Public Dataset

Initially, we examined the GEO public dataset (GSE104687) (https://www.ncbi.nlm.nih.gov/geo) comprising 376 samples from cortical gray (parietal and temporal) and white matter (parietal) as well as hippocampal tissues gathered from 107 brains. The collected data underwent meticulous normalization and subsequent analysis to identify disparities in CDHR1 expression across distinct tissues, namely the hippocampus, white matter of the forebrain, parietal neocortex, and temporal neocortex, within both the control and TBI groups (Miller et al. 2017).

Information pertinent to inflammation- and metabolism-related pathways was sourced from the Molecular Signature Database, MSigDB (Rosario et al. 2018). Gene Set Variation Analysis was performed to compute the enrichment scores, and Pearson’s correlation analysis was employed to assess the relationship between CDHR1 expression and these pathways. Additionally, the xCell algorithm (Aran et al. 2017) was used to estimate the distribution of immune cells within the TBI samples, and Pearson correlation analysis was implemented to discern its correlation with CDHR1.

### Integration of Genetic Association Analysis

Subsequently, genetic association analysis was conducted by integrating the genome-wide association study (GWAS), expressed quantitative trait loci (eQTL), and methylation quantitative trait loci (mQTL) data. GWAS summary data for TBI were obtained from the publicly accessible OpenGWAS database (https://gwas.mrcieu.ac = /) under the code name finn-b-TRAUMBRAIN_NONCONCUS, encompassing 3193 patients within the European population, involving 215,599 cases and controls. Blood eQTL data were acquired from the BrainMeta dataset resulting from a meta-analysis of the GTEx brain (GTEx Consortium 2020), CMC (Fromer et al. 2016) and ROSMAP (Ng et al. 2017) by meta-analysis of cis-eQTLs in correlated samples in the study of genetic regulation differences in gene expression between the brain and blood, as summarized by Qi et al. (2018). The mQTL data originated from the LBC-BSGS blood mQTL dataset (*n* = 1980), a compilation of two independent datasets: the Brisbane Systems Genetics Study (*n* = 614) (Powell et al. 2012) and the Lothian Birth Cohort (LBC, *n* = 1366) (Chen et al. 2016). Moreover, for brain tissue eQTL analysis, data from the GTEx Consortium (2020) were used to explore genetic associations and gene expression across 49 tissues sourced from 838 humans.

### SMR and HEIDI Analysis

SMR and HEIDI analyses were performed using summary-level data from GWAS and eQTL studies. These analyses aimed to identify pleiotropic associations between gene expression levels and complex traits, as well as to assess whether SNP effects on phenotypes were mediated by gene expression. In a multi-omics context integrating summary-level data from blood eQTL and mQTL, SMR prioritized potential genes associated with GWAS for subsequent investigations. HEIDI testing was performed to evaluate pleiotropic effects and cascading imbalances.

This analysis was stratified into three distinct steps:

1. SMR analysis was conducted using single-nucleotide polymorphism (SNPs) as genetic instruments, CDHR1 transcript in blood as the exposure, and TBI as the outcome.
2. SMR analysis was repeated employing SNPs as genetic instruments, CDHR1 methylated expression in blood as the exposure, and TBI as the outcome.

Criteria for SMR and HEIDI tests encompassed *P*_HEIDI_ > 0.01 and FDR > 0.05, with FDR calculated using the fdr algorithm and Bonferroni correction for multiple comparisons.

### Mouse Source and TBI Modeling

Adult C57BL/6J mice (males, 6–8 weeks old, weighing 16–20 g) were procured from GEMPHARMATECH Co., Ltd. (Jiangsu, China) and housed under strict specific-pathogen free conditions with access to sterilized food and water. All experimental procedures were conducted in compliance with the National Institutes of Health guidelines for laboratory animals and were supervised and approved by the Ethics Committee of Nanchang University (Ethics Approval Number NCULAE-20221108001).

The TBI model employed in this study was executed two weeks after the mice were acclimatized. Anesthesia was induced by an intraperitoneal injection of sodium pentobarbital (60 mg/kg). Subsequently, mice were securely fixed, shaved, disinfected, and subjected to skull stripping. In the TBI group, the left parietal bone was exposed by drilling 3.5 mm posteriorly and 2.5 mm laterally to the anterior fontanelle. A spacer was placed on the dura mater, and a 20 g weight was released from a height of 25 cm, thereby inducing injury. In contrast, the sham group underwent skull exposure only. Following this procedure, all experimental mice were housed individually in suitable environments.

### Western Blotting

On the third day post-injury, the TBI (*n* = 5) and sham (*n* = 5) mice were euthanized under anesthesia induced by sodium pentobarbital. The cortex (2 × 2 × 2 mm) from the impact area and its peripheral region, along with the hippocampus from the impact side, were carefully dissected. Approximately, 10 μg of tissue was homogenized at 4 °C in 200 μl of RIPA lysate. Protein concentrations were determined using a BCA kit and absorbance was measured at 562 nm using a spectrophotometer. Electrophoresis was performed on 8–15% SDS-PAGE gels at 80–120 V, and subsequent protein transfer onto a 0.45 μm PVDF membrane occurred at 280 mA for 1 h. Blocking was performed using 5% BSA for 2 h. Primary antibodies including β-actin (1:20,000, Cat. 66009-1-Ig; Proteintech, China) and CDHR1 (1:2000, Cat. AP55261; Abcam) overnight at 4 °C. Secondary antibodies (1:10,000, goat anti-mouse IgG H&L (Cat. ab205719; Abcam, USA), and goat anti-rabbit IgG H&L (1:10,000; Cat. ab205718; Abcam, Cambridge, USA) for 1–2 h. Membranes were washed thoroughly with TBST and then incubated with ECL exposure solution, followed by development under a chemiluminescence system (GEAI600, MA, USA) under light-protected conditions. Quantitative analyses were performed using Quantity One (version 4.6.6).

### Immunohistochemistry

Brain tissues including the cortex and CA1 region of the hippocampus from both sham and TBI mice were collected, embedded in paraffin, and sectioned into 4 μm thick slices. Prior to staining, the sections were dewaxed, hydrated, and subjected to sodium citrate antigen retrieval, and serum was blocked. Incubation with primary antibodies against CDHR1 (1:2000, Cat. AP55261; Abcam) overnight at 4 °C. Following adequate washing, secondary antibody (1:1000, Cat. ab150176, Goat Anti-Chicken IgY H&L, Alexa Fluor® 594, Abcam, USA) incubation ensued. DAPI (1:10,000, Beyotime Institute of Biotechnology) was used for nuclear counterstaining. Images were captured using a fluorescence microscope (Leica, China) and assessed using the ImageJ software.

### Detection of Expression Levels of Inflammatory Markers

Mice from the sham and TBI groups were euthanized on the third day and hippocampal and cortical tissues were carefully separated. Hippocampal tissues were lysed using RIPA and protease inhibitors, and tissues were homogenized using ultrasound. The tissue was centrifuged at 12,000 × *g* for 10 min at 4 °C and the supernatant was collected in a 1.5 ml centrifuge tube for subsequent analysis. Step by step, TNF-β1, IL-10, IFN-γ, and IL-6 expression levels were detected according to the instructions of the enzyme-linked immunosorbent assay (ELISA) kit (both purchased from Thermo Fisher Scientific, United States).

### Statistical Analysis

Basic statistical analyses were conducted using R (version 4.1.3, https://www.r-project.org) and GraphPad Prism 8 software (GraphPad Software, Inc., San Diego, CA, USA). Data are presented as mean ± standard deviation (SD). Statistical significance was set at *p < 0.05. The normality of data distribution within each group was evaluated using the Shapiro–Wilk test, and homogeneity of variance was tested using Levene's tests. If the data were not normally distributed or the variance was not heterogeneous, nonparametric tests were used. A Student’s *t*-test was performed to compare the two groups. SMR (https://cnsgenomic.com/software/smr/) was employed for correlation analysis between the multi-cohort data and GWAS. All data were derived from at least three independent trials. All the experiments and statistical analyses were blinded.

## Results

### CDHR1 Expression in Brain Tissues of Patients with TBI

**Fig. 1:**
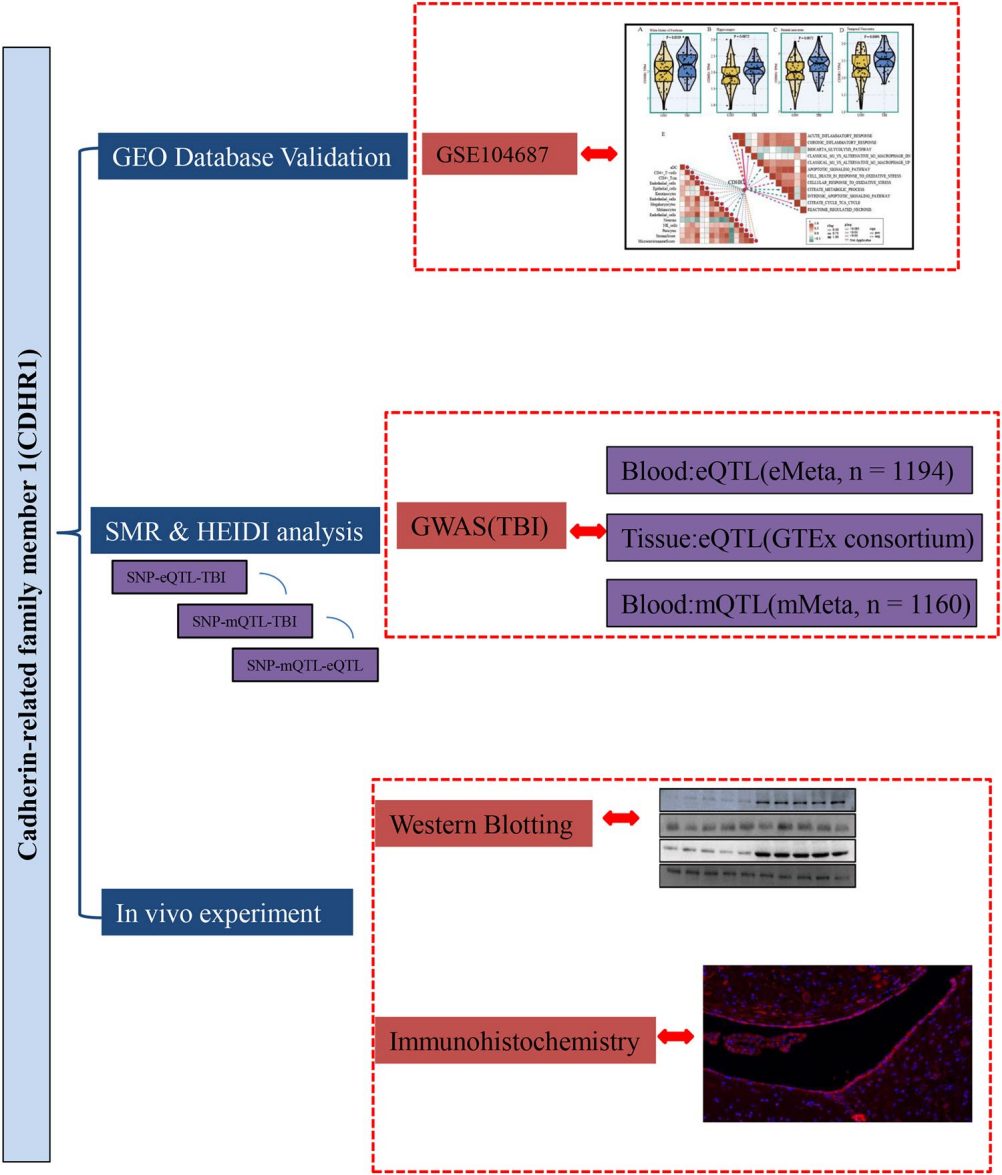
Flowchart of the study

**Fig. 2:**
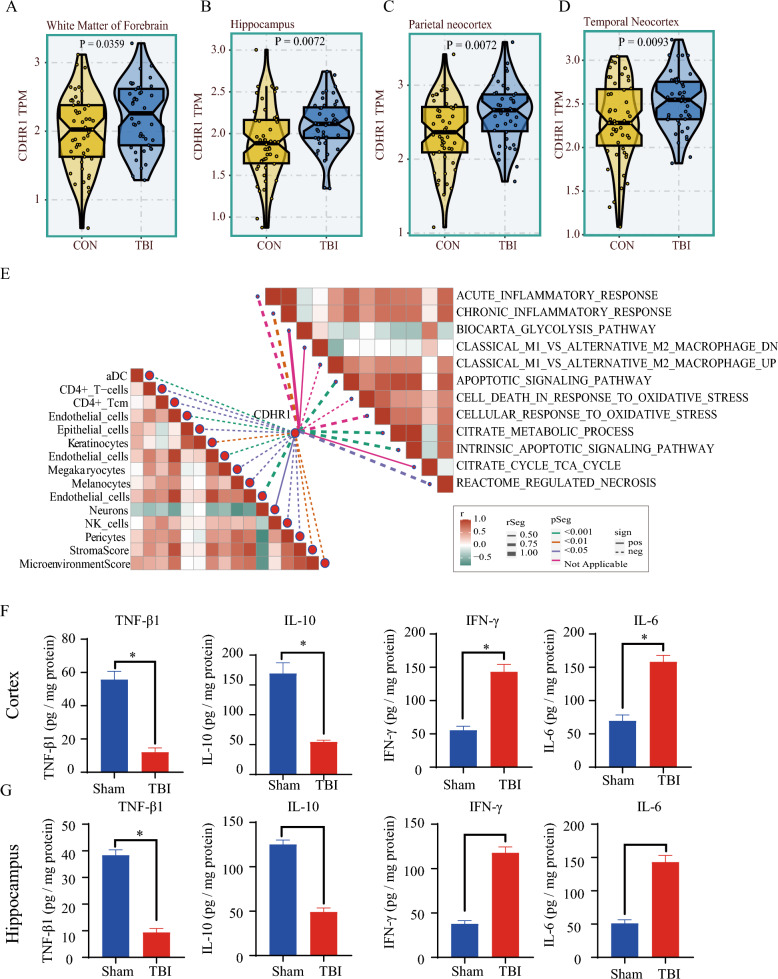
Comprehensive analysis was conducted using the public database GSE104687, encompassing distinct brain regions and detection of pro-inflammatory and anti-inflammatory cytokines through enzyme-linked immunosorbent assay (ELISA): **A** white matter of forebrain, **B** hippocampus, **C** parietal neocortex, **D** temporal neocortex, **E** the left side illustrates correlation analyses between immune cells and CDHR1, employing xCell methodology, while the right side presents correlation analyses between inflammatory/metabolic pathways and CDHR1. Pathway enrichment was assessed through Gene Set Variation Analysis, as described in the “Methods” section. **F** In cortical tissue, pro-inflammatory factors (TNF-β1 and IL-10) and anti-inflammatory factors (IFN-γ and IL-6) were detected by ELISA, and **G** hippocampal tissue, pro-inflammatory factors (TNF-β1 and IL-10), and the expression levels of anti-inflammatory factors (IFN-γ and IL-6). The bar graph is presented as mean ± standard deviation (*n* = 3, number of animals); *p < 0.05 vs. sham group

Figure 1 illustrates the study design. We meticulously examined the CDHR1 transcriptome using a publicly accessible dataset (GSE104687). This dataset included 55 participants with TBI who reported loss of consciousness and were meticulously matched with 55 control patients based on age, sex, and year of death. The participants provided brain samples from various regions, including the hippocampus, white matter of the forebrain, parietal neocortex, and temporal neocortex, totaling 376 samples. Our differential analysis of CDHR1 expression across different brain tissues revealed significantly elevated levels compared to those in the control group (white matter of the forebrain, *p* = 0.0359; hippocampus, *p* = 0.0072; parietal neocortex, *p* = 0.0072; and temporal neocortex, *p* = 0.0093, Fig. 2A–D).

Moreover, when examining the correlation between CDHR1 expression and pathways related to immune cells and inflammation, we observed a robust association between CDHR1 expression and endothelial cells. Notably, acute and chronic inflammatory processes have emerged as pivotal explanations for TBI pathogenesis of TBI, underscoring the significance of CDHR1 in these mechanisms (Fig. 2E).

We examined the levels of pro-inflammatory factors (TNF-β1 and IL-10) and anti-inflammatory factors (IFN-γ and IL-6). In cortical tissues, the concentrations of TNF-β1 (unpaired *t*-test, *t* = 13.30, df = 4, *p* = 0.0002) and IL-10 (unpaired *t*-test, *t* = 10.73, df = 4, *p* = 0.0004) were significantly higher in the TBI group compared to the sham group. For the anti-inflammatory factors, the concentrations of IFN-γ and IL-6 were both correspondingly decreased (sham vs. TBI, unpaired *t*-test, *t* = 11.88, df = 4, *p* = 0.0003; sham vs. TBI, unpaired *t*-test, *t* = 11.47, df = 4, *p* = 0.0003, Fig. 2F). In mouse hippocampal tissues, pro-inflammatory factors (TNF-β1, sham vs. TBI, unpaired *t*-test, *t* = 19.45, df = 4, *p* < 0.0001; IL-10, sham vs. TBI, unpaired *t*-test, *t* = 19.41, df = 4, *p* < 0.0001) and anti-inflammatory factors (IFN-γ, sham vs. TBI, unpaired *t*-test, *t* = 17.79, df = 4, *p* < 0.0001; IL-6, sham vs. TBI, unpaired *t*-test, *t* = 13.77, df = 4, *p* = 0.0002) showed similar performance (Fig. 2G). This suggests that TBI occurs throughout the course of inflammation.

### Incorporating Associations Between Multi-omics Data from Blood and TBI

**Fig. 3:**
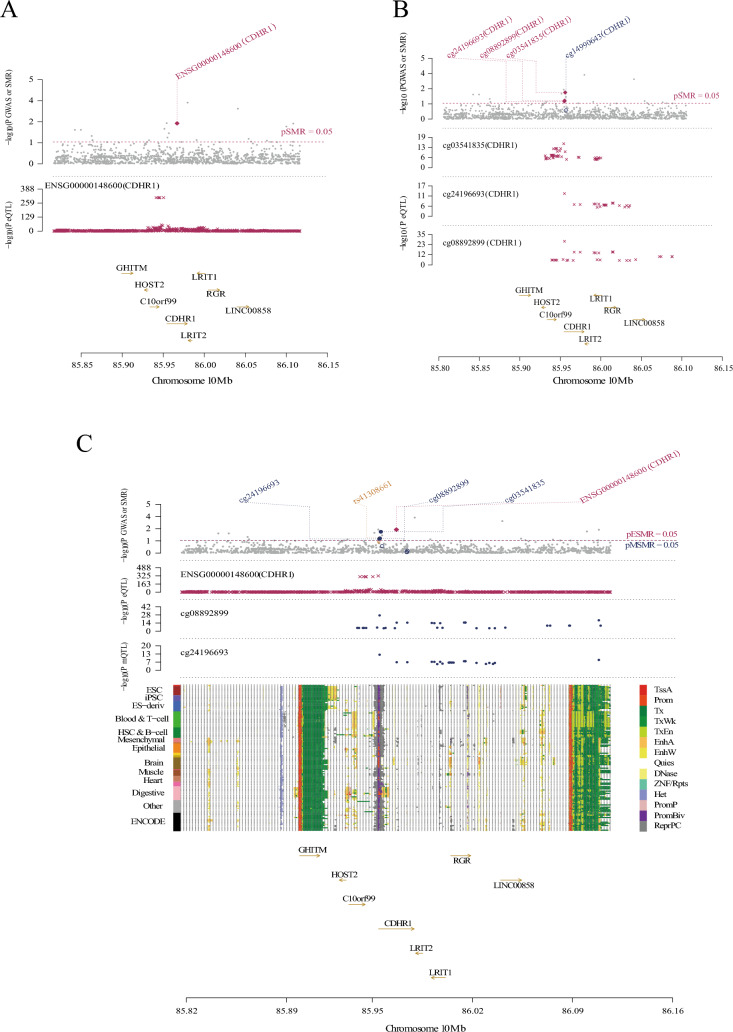
Association of CDHR1 with traumatic brain injury (TBI) in blood. **A** eMeta dataset. Solid diamonds represent probes that passed the HEIDI test, while hollow diamonds indicate probes that failed the HEIDI test. Probes highlighted in maroon passed the Summary-data-based Mendelian Randomization (SMR) threshold; **B** similar to the eMeta dataset, the LBC_BSGS dataset shows associations consistent with the eMeta dataset, and **C** single nucleotide polymorphisms (SNP) and SMR association results of methylation quantitative trait loci (mQTL), expressed quantitative trait loci (eQTL), and genome-wide association study (GWAS). The first graph illustrates the SNP-log_10_ (*p*-value) of TBI from GWAS. Red diamonds and blue diamonds depict − log_10_ (*p*-value) in the SMR test for the association of CDHR1 expression and methylation probes with TBI, respectively. Solid diamonds denote probes that passed the HEIDI test, whereas hollow diamonds represent probes that did not pass the HEIDI test. Yellow asterisks indicate the top SNP

Based on the aforementioned finding of differential CDHR1 expression in TBI, we hypothesized that CDHR1 expression could explain the causal presumption of the disease. Our objective was to delineate the gene regulation of CDHR1 in blood during TBI and explore the potential underlying epigenetic regulatory mechanisms. To achieve this, we employed a three-step SMR approach. Instances where HEIDI > 0.05 were considered to lack significant heterogeneity. This study integrated cis-eQTL and cis-mQL data for CDHR1 and amalgamated them with GWAS summary-level statistics from the largest TBI dataset (Fig. 3A).

Specifically, in the GWAS summary statistics analysis from BrainMeta (*n* = 1194) and TBI, we integrated the results of eQTL for CDHR1, focusing on probes ENSG00000148600 for CDHR1 (β-SMR = 0.0603821, p-SMR = 0.003934558, p-HEIDI = 0.7282966, and FDR = 0.00393). Similarly, we incorporated summary-level data of mQTL from the LBC-BSGS (*n* = 1980) to identify probes related to the epigenetic inheritance of CDHR1. Our analysis identified eight methylation sites, of which cg24196693, cg08892899, and cg03541835 were the most prominent (p-SMR < 0.05, p-HEIDI > 0.05). Following FDR correction, we selected cg03541835 as the prime CpG site candidate (β-SMR = 0.231634, p-SMR = 0.006528648, p-HEIDI = 0.2205731, and FDR = 0.052229184) despite an FDR exceeding 0.05 (Fig. 3B).

These results underscored the association between SNPs linked to CDHR1 in GWAS and eQTL as well as GWAS and mQTL of TBI (Fig. 3C). The expression level of CDHR1 (β-SMR = 0.0603821) exhibited a positive correlation with the onset of TBI, mirroring the analogous relationship observed in the level of methylation at the cg03541835 site (β-SMR = 0.0603821). In conclusion, our study elucidated a mechanism explaining the causal effect of CDHR1 in the presumptive occurrence of TBI in the blood, which is potentially influenced by both CDHR1 transcriptional expression and epigenetic modifications.

The second graph shows the − log_10_ (*p*-value) of the SNP association of the CDHR1 gene probe ENSG00000148600 from the eMeta dataset. The third graph presents the association of SNPs [− log_10_ (*p*-value)] with methylation probes from the LBC_BSGS dataset.

In the lower panel, 127 chromatin state annotations (in color) for 14 samples from the Roadmap Epigenomics Mapping Consortium (REMC) are shown for different primary cell and tissue types (rows). REMC = Roadmap Epigenomics Mapping Consortium, TSSA = active Transcription Start Site, Prom = promoter, and Tx. for active transcription; TxWk for weak transcription; TxEn for transcribed and regulatory promoters/enhancers; EnhA for active enhancers; and EnhW for weak enhancers. Other annotations include DNase (primary DNase), ZNF/Rpts (ZNF genes and repeats), Het (heterochromatin), PromP (poised promoter), PromBiv (bivalent promoter), ReprPC (repressed PolyComb), and Quies (quiescent/low).

### Integration of GWAS and eQTL Data from Brain Tissue

**Fig. 4:**
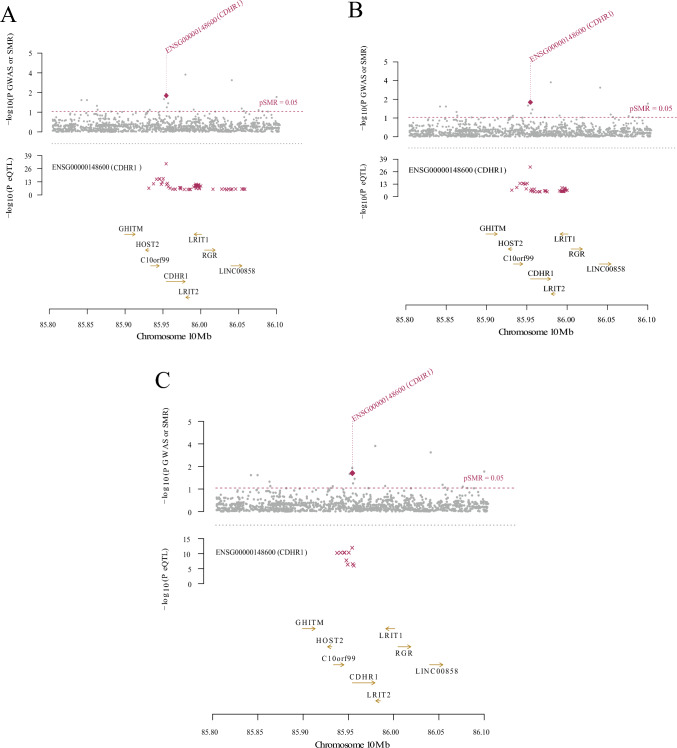
Association between CDHR1 and traumatic brain injury in different tissues. **A** Cortex, **B** frontal cortex, and **C** hippocampus. Solid diamonds indicate probes that passed the HEIDI test and hollow diamonds indicate probes that failed the HEIDI test. Labels highlighted in maroon indicate probes that passed the Summary-data-based Mendelian Randomization threshold

Gene expression patterns vary significantly between blood and tissue locations, with the genetic heterogeneity of TBI contributing to these differences. We hypothesized that understanding the interplay between eQTL expression in the brain tissue and TBI could help elucidate causal inferences related to TBI. To test this, we selected cis-eQTL data from the cortex, hippocampus, and frontal cortex of the GTEx Consortium. Subsequently, these datasets were subjected to SMR analysis within the cortex (p-SMR = 0.005003246, p-HEIDI = 0.5233138, and FDR = 0.005003246, Fig. 4A), frontal cortex (p-SMR = 0.00500549, p-HEIDI = 0.5401322, and FDR = 0.005005, Fig. 4B), and hippocampus (p-SMR = 0.007399831, p-HEIDI = 0.1626091, and FDR = 0.007399831, Fig. 4C).

The SMR analysis yields compelling results. Notably, CDHR1 exhibited a significant absence of association within the cortex (β-SMR = 0.0812947), frontal cortex (β-SMR = 0.0895947), and hippocampus (β-SMR = 0.140338). However, the expression of CDHR1 in the frontal cortex, hippocampus, and cortex may play a causal role in TBI (Fig. 4).

### Validation of CDHR1 in an In Vivo Model of TBI

**Fig. 5:**
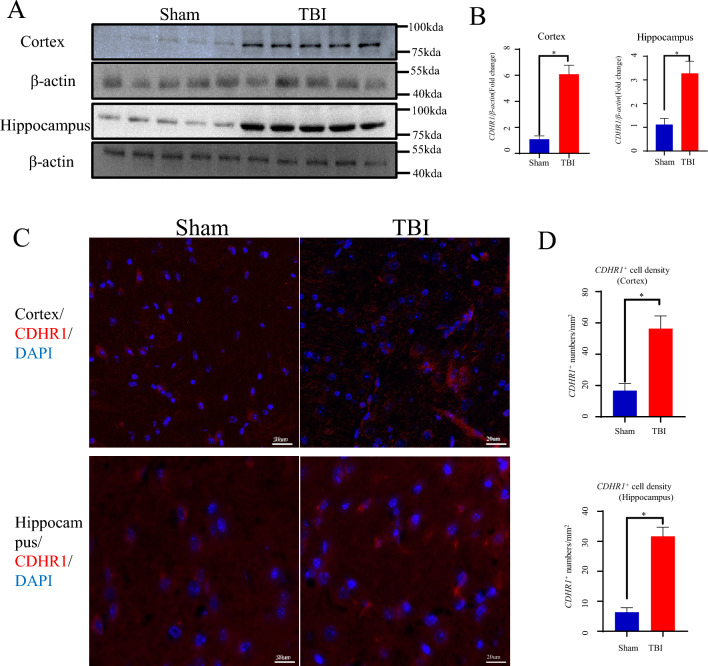
CDHR1 expression levels detected in an in vivo traumatic brain injury (TBI) model. **A**, **B** Comparative analysis of CDHR1 expression levels in distinct brain tissues (TBI vs. sham group), encompassing the cortex and hippocampus, and **C**, **D** Furthermore, fluorescence microscopy was employed to examine the fluorescence expression and quantification of CDHR1 in different brain tissues (TBI vs. sham group). The bar graph is presented as mean ± SD (*n* = 5, number of animals); *p < 0.05 vs. sham group

In this study, we utilized a TBI model induced by gravity strikes and examined the cortical and hippocampal tissues. Through rigorous analyses using western blotting and immunohistochemistry, we elucidated the expression patterns of CDHR1. Our findings revealed a significant upregulation of CDHR1 protein expression in both the cortex (unpaired *t*-test, *t* = 15.25, df = 8, *p* < 0.0001) and the hippocampus (unpaired *t*-test, *t* = 8.485, df = 8, *p* < 0.0001) when compared with the sham group, demonstrating a consistent trend (Fig. 5A, B). This pattern was further corroborated by immunohistochemical analysis, underscoring the robustness of our results (cortex: TBI vs. sham, unpaired *t*-test, *t* = 8.929, df = 4, *p* = 0.0009; hippocampus: TBI vs. sham, unpaired *t*-test, *t* = 8.332, df = 4, *p* = 0.0011, Fig. 5C, D). We also examined the contralateral side and the expression of TBI of different severities. Thus, our data unequivocally established a direct association between CDHR1 expression and TBI.

These results lead us to posit that heightened CDHR1 expression observed in the context of TBI may serve as a pivotal target for potential therapeutic interventions or diagnostic strategies.

## Discussion

In this study, we meticulously examined the expression levels of CDHR1 across various brain tissues using a public GEO dataset. Subsequently, we investigated the potential causal relationship between CDHR1 and TBI across blood and different tissues. Through an integrative analysis encompassing GWAS, eQTL, and mQTL associations using the SMR approach, we identified a positive correlation between CDHR1 transcripts and methylation sites and the risk of TBI. Additionally, by developing a TBI model, we scrutinized the expression levels and fluorescence quantification of CDHR1 in cortical and hippocampal tissues. Our findings highlight a possible regulatory link between CDHR1 and TBI from diverse perspectives, offering innovative avenues for exploring the mechanisms underlying TBI.

TBI manifests as a swiftly progressive ailment, with some patients exhibiting rapid responses within weeks, and others enduring prolonged complications. Researchers have extensively probed genetic variability using genetic polymorphisms that exacerbate TBI outcomes (Reddi et al. 2022; Jordan 2007). Notably, individuals carrying the ε4 allele following mild TBI exhibited diminished neuropsychological outcomes compared to those lacking this allele (Liberman et al. 2002). Beyond tau, the neurotrophic factor brain-derived neurotrophic factor also plays a pivotal role in TBI. Genetic studies have underscored their significance, paving the way for precise treatments tailored to patients with TBI.

Although CDHR1 has undergone extensive scrutiny within the context of retinal dystrophy, where mutations in CDHR1 hold potential therapeutic promise, and subtype alleles associated with CDHR1-linked retinitis pigmentosa variants have been identified (Malechka et al. 2022; Stingl et al. 2017), its exploration within the realm of TBI remains limited.

Here, we examined the results derived from the differential expression analysis of CDHR1 across various brain tissues sourced from the GEO dataset GSE104687. This comprehensive analysis not only provides compelling evidence regarding the pivotal role of CDHR1 in TBI but also contributes significantly to the expanding body of knowledge in this domain.

The current landscape of scientific inquiry has witnessed a growing emphasis on the development of blood-based markers for the detection of physiological activity in TBI, both in the realms of research and clinical application. The exploration of disease-specific markers within the blood milieu holds promise for delving deeper into the intricate fabric of disease. In this study, we postulated a causal nexus between CDHR1 and TBI. To substantiate this hypothesis, we performed SMR analyses of publicly available blood sources, targeting the transcriptome and epigenetic signatures within the bloodstream. Our approach, akin to pioneering studies in related fields, harnessed the power of genetic variation to simulate potential exposure factor–gene relationships. Krishnamoorthy et al. analyzed transcriptomic data encompassing 49 tissues, illuminating specific genes associated with COVID-19 and SARS-COV-19 (Krishnamoorthy et al. 2023). Their discoveries not only deepened our understanding of these diseases but also laid a foundation for prospective therapeutic drug development. Similarly, Liu et al. discovered a mechanism mediated by genetic variation, shedding light on the intricate interplay between genetic regulation and methylation sites in AF (Liu et al. 2021). In our study, we discerned a noteworthy connection between increased CDHR1 transcription (β-SMR = 0.0603821) and heightened TBI risk, hinting at a significant correlation between the transcriptome, methylation patterns, and TBI. This finding was confirmed by our analysis of high-CDHR1 expression in the TBI group, which is concordant with our GEO data analysis.

The exploration of disease-specific tissues offers a novel vantage point for unraveling the molecular intricacies underlying afflictions. Here, we used brain tissue eQTL analyses to determine the genetic impact of CDHR1 expression on TBI. Given the acute nature of TBI and its direct impact on brain tissues, alterations in genes within these tissues are key to elucidating the underlying biomolecular mechanisms. Through our application of SMR, we probed the association of eQTL for CDHR1 in both the cortex and hippocampus with TBI. CDHR1, a vital member of the cadherin superfamily that is intricately woven into neural circuits, human auditory neurodevelopment, and neuronal connectivity interactions, emerged as the focal point of our study. Our findings underscore the heightened risk of TBI associated with elevated CDHR1 expression, a trend observed in both the cortex and the hippocampus. Complementary investigations in a mouse model of TBI corroborated our findings, indicating robust CDHR1 expression in the cortical and hippocampal tissues. Nevertheless, a deeper exploration of the genetic background is imperative to elucidate the critical role of CDHR1 in TBI.

One of the strengths of our study is the adept integration of data sourced from the GEO transcriptome. Through this integration, we dissected the mechanisms underlying TBI across diverse histological contexts, encompassing both blood and brain tissues. This is the first study based on an expansive TBI GWAS dataset that specifically delves into the intricacies of CDHR1. Our study not only offers invaluable insights from the realms of genetics and genoproteomics, but also underwent rigorous validation through in vivo experiments, thereby bolstering the credibility of our conclusions.

However, this study also had some limitations. Our focus was primarily on the cis-region of CDHR1, warranting further exploration of its broader genetic landscape. Additionally, it is imperative to recognize that GWAS-based investigators are predominantly of European origin, which potentially limits the generalizability of our findings, particularly in regions such as Asia. Furthermore, the prospect of conducting knockout studies promises a more nuanced understanding of the intricate web of interactions involving CDHR1 in TBI. Finally, although we obtained published data to analyze the differential expression of CDHR1 in different tissues and its causal association in different tissues, the corresponding tissues lacked clinical information on the patients, which may have obscured some important information.

In summary, our study represents a significant step in TBI research by shedding light on the multifaceted interplay between CDHR1 and this debilitating condition. Our findings not only enrich the current scientific discourse but also pave the way for future investigations aimed at illustrating the complexities of TBI at the molecular level.

## Conclusion

We demonstrated the association between the CDHR1 integrated genome, transcriptome, and epigenetics and TBI based on various histological approaches and that CDHR1 may have a biological role in TBI and may serve as a promising option for the clinical treatment of TBI.

## Supplementary Information

Below is the link to the electronic supplementary material.

Supplementary file1 (DOCX 5634 kb)

## Data Availability

Data supporting the findings of this study are available from the Massachusetts Institute of Technology (MIT) and Beth Israel Deaconess Medical Center (BIDMC); however, limitations apply to the availability of these data, which were used with permission from the current study and are therefore not public. However, the data are available to the authors upon reasonable request and with permission from the Massachusetts Institute of Technology (MIT) and the Beth Israel Deaconess Medical Center (BIDMC).

## Declarations

### Research Involving Human and/or Animal Participants

This study did not include any research conducted on human or animal participants.
