# Supplementary material for: Association of Cadherin-Related Family Member 1 with Traumatic Brain Injury
Source: Cell Mol Neurobiol. 2024 Apr 24;44:41. doi: 10.1007/s10571-024-01476-3 (PMC11043179; doi:10.1007/s10571-024-01476-3)

**Supplementary Information**

**Journal name:** **Cellular and Molecular Neurobiology**

**Association of cadherin-related family member 1 with traumatic brain injury**

Yong’An Jiang^1,2,*^, Peng Chen^1,2,*^, YangYang Zhao^1,2,*^, Yan Zhang^1,#^

**Authors:**

Yong’An Jiang^1,2^

^1^Department of Neurosurgery.The Second Affiliated Hospital of Nanchang University. Nanchang ,330008, Jiangxi, P. R. China.

^2^Nanchang University, Nanchang ,330008, Jiangxi, P. R. China.

**Email:** [jiangya@email.ncu.edu.cn](mailto:jiangya@email.ncu.edu.cn)

**ORCID:**0000-0001-5337-1786

Peng Chen^1,2,*^

^1^Department of Neurosurgery.The Second Affiliated Hospital of Nanchang University. Nanchang 330006, Jiangxi, P. R. China.

^2^Nanchang University, Nanchang ,330006, Jiangxi, P. R. China.

**Email:**cpneuro@163.com

**ORCID:** 0000-0002-1536-4945

Yangyang Zhao^1,2,*^

^1^Department of Neurosurgery.The Second Affiliated Hospital of Nanchang University. Nanchang 330006, Jiangxi, P. R. China.

^2^Nanchang University, Nanchang ,330006, Jiangxi, P. R. China.

**Email:** [zhaoyangyang@email.ncu.edu.cn](mailto:zhaoyangyang@email.ncu.edu.cn)

**ORCID:** 0000-0003-0184-9244

Yan Zhang^1,#^

^1^Department of Neurosurgery.The Second Affiliated Hospital of Nanchang University. Nanchang 330008, Jiangxi, P. R. China.

**Email:** ndefy12388@ncu.edu.cn

**ORCID:**0009-0008-3525-6272

**Correspondence^#^:**

Yan Zhang^1,#^

^1^Department of Neurosurgery.The Second Affiliated Hospital of Nanchang University. Nanchang 330008, Jiangxi, P. R. China.

**Email:**d ndefy12388@ncu.edu.cn

**ORCID:**0009-0008-3525-6272

Yong’An Jiang^1,2,*^, Peng Chen^1,2,*^ YangYang Zhao^1,2,*^ have contributed equally to this work

**Fig.S1**
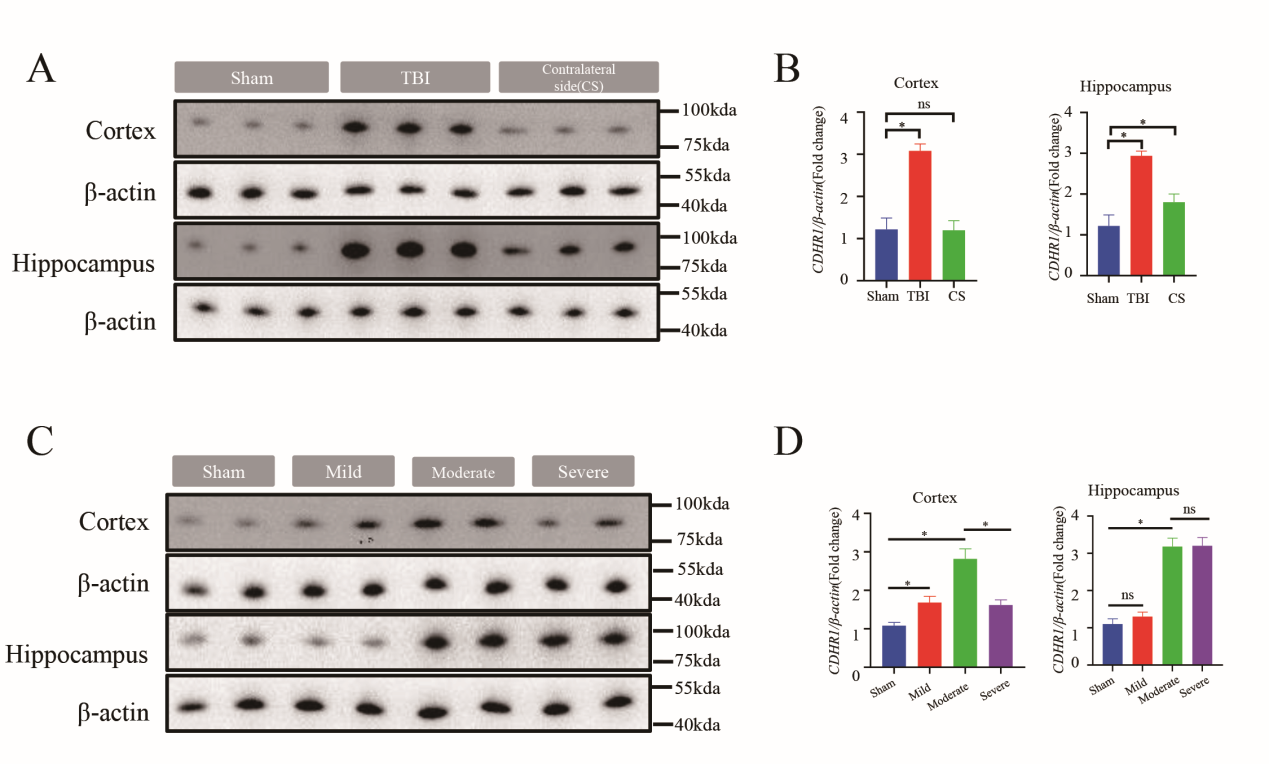


**Fig.S1** Expression levels of CDHR1 in different brain tissue locations and disease severity. (a-b) Comparative analysis of CDHR1 expression levels in ipsilateral and contralateral brain tissues (traumatic brain injury vs. sham group), encompassing the cortex and hippocampus. (c-d) CDHR1 expression levels in the sham group, mild group, moderate group, and severe group. the bar graph is presented as mean ± standard deviation (n = 3，number of animals); ^*^p <0.05 vs. sham group.

Figure 5A cortex_CDHR1


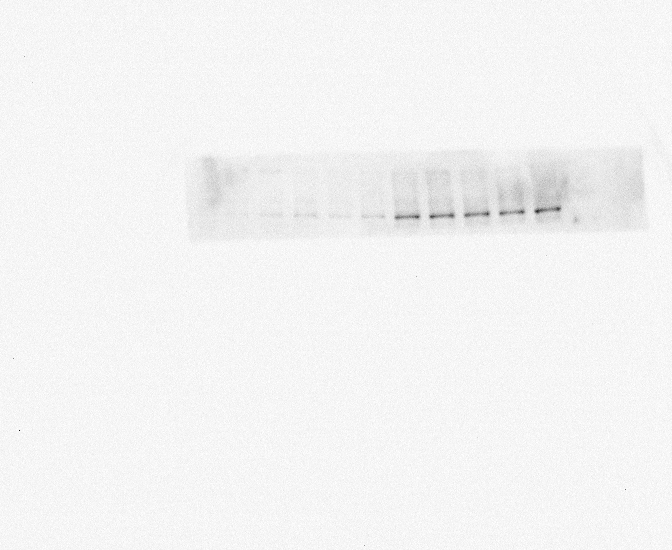


Figure 5A cortex_actin


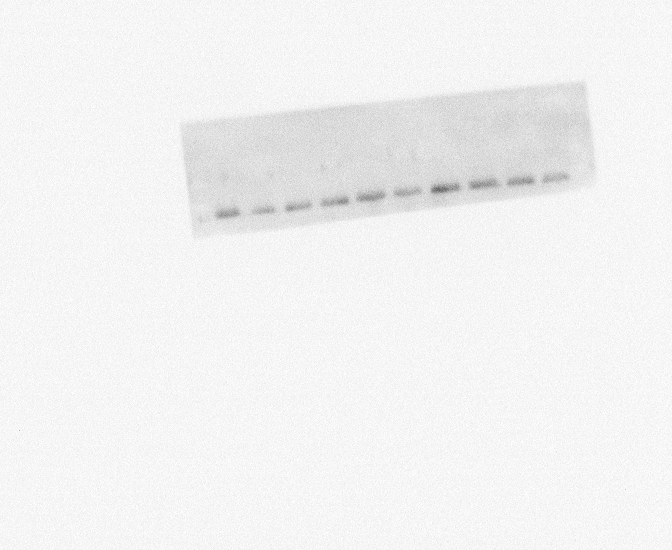


Figure 5A Hippocampus_CDHR1


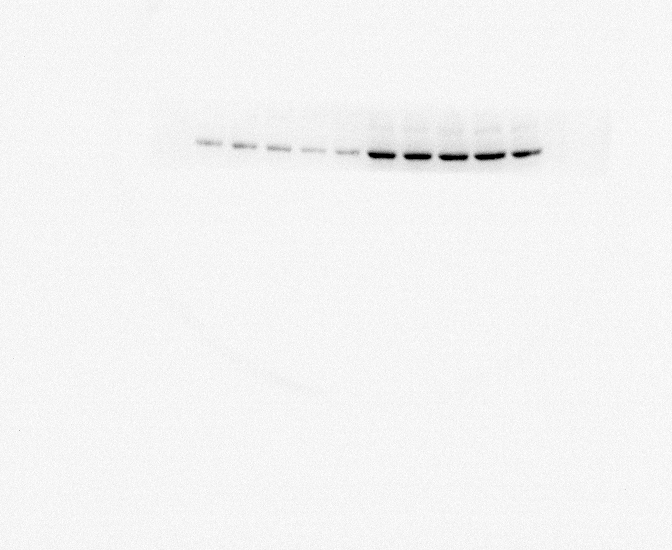


Figure 5A Hippocampus_β-actin


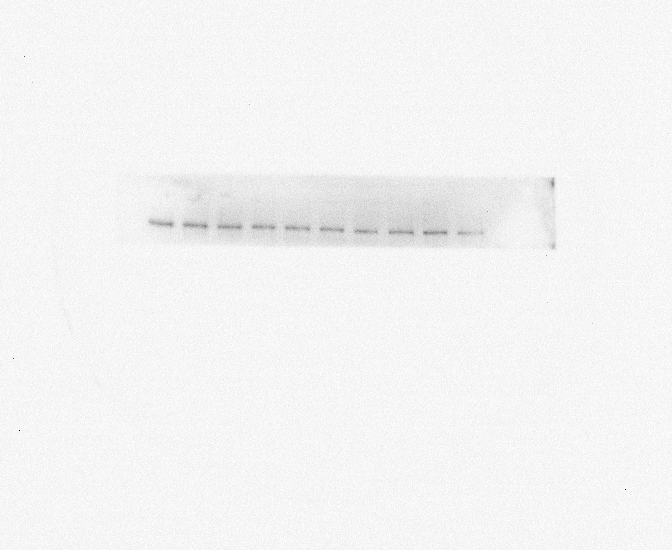


Figure S1A_cortex_CDHR1


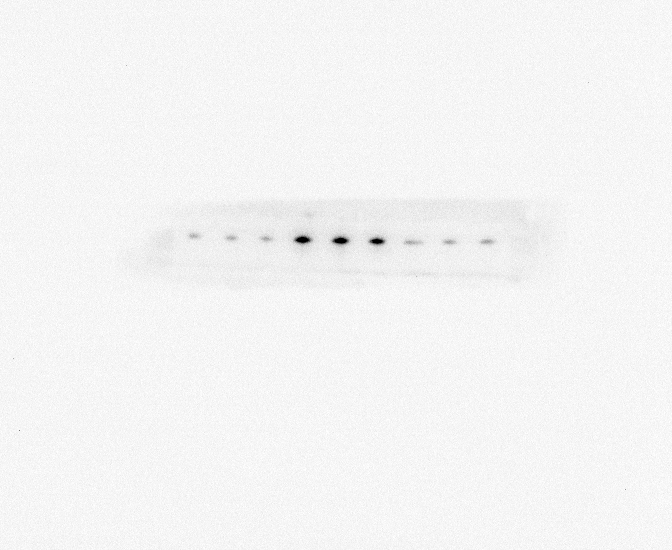


Figure S1A_cortex_β-actin


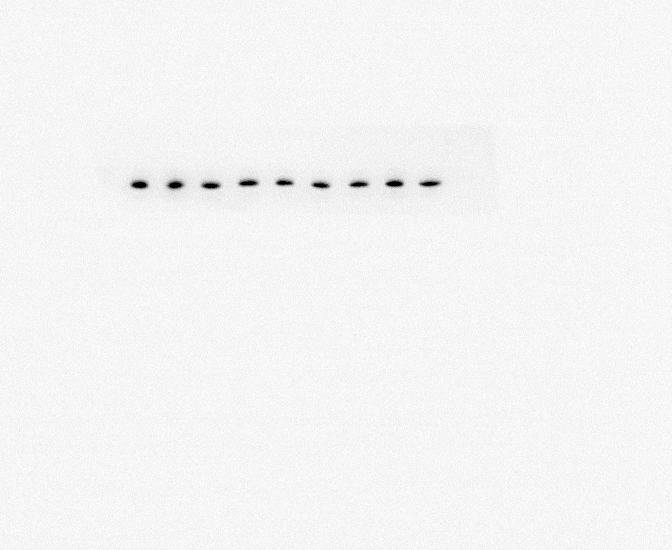


Figure S1A_hippocampus_CDHR1


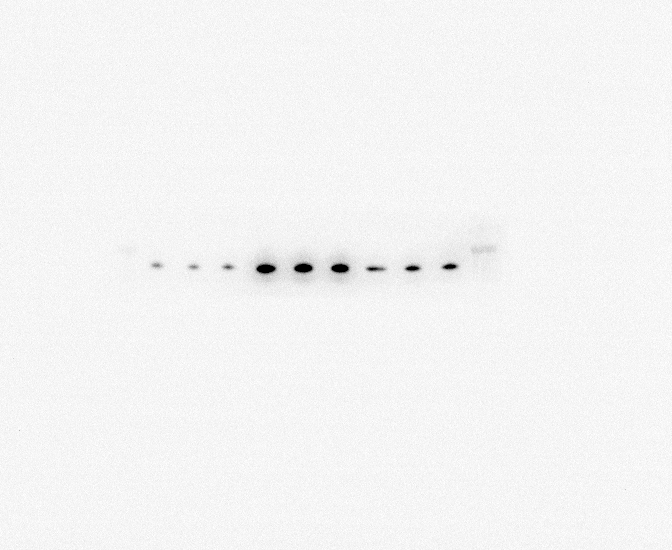


Figure S1A_hippocampus_β-actin


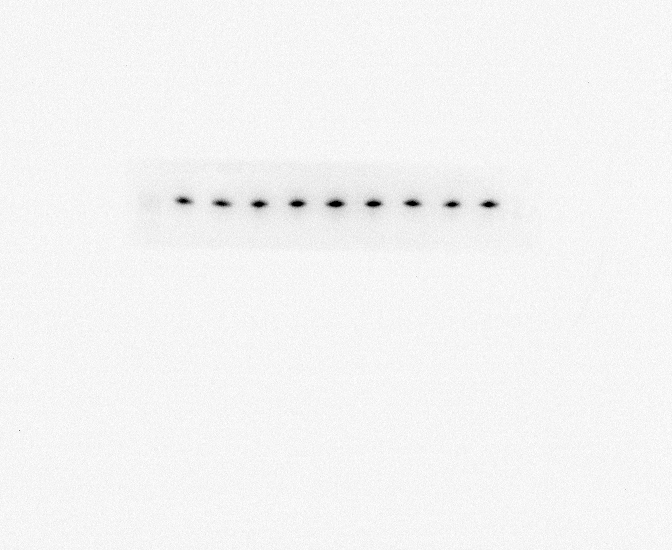


Figure S1B_cortex_CDHR1


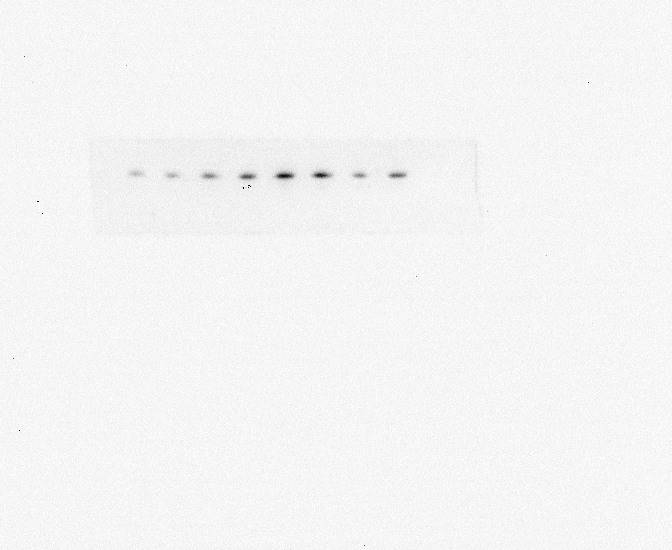


Figure S1B_cortex_β-actin


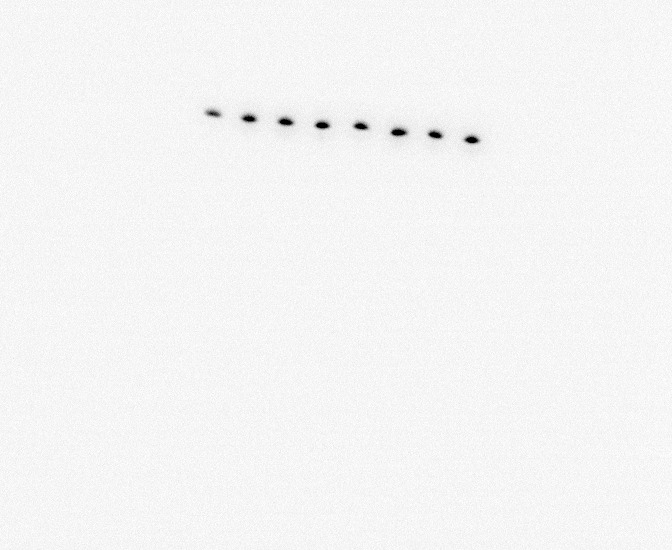


Figure S1B_hippocampus_CDHR1


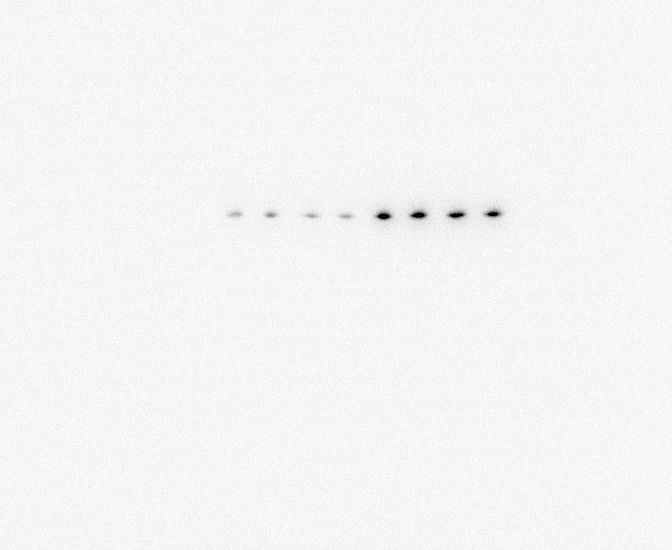


Figure S1B_hippocampus_β-actin


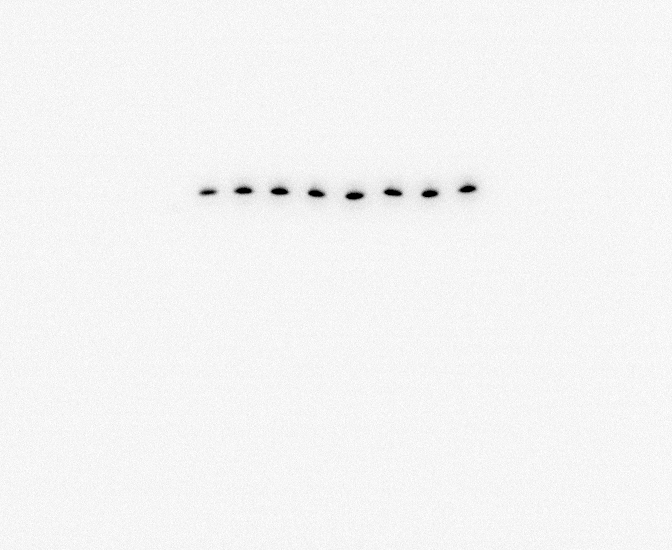

Supplement: Supplementary file 1 — Supplementary file1 (DOCX 5634 kb) [file 10571_2024_1476_MOESM1_ESM.docx]
